# LPI Radar Waveform Recognition Based on Neural Architecture Search

**DOI:** 10.1155/2022/4628481

**Published:** 2022-01-18

**Authors:** Zhiyuan Ma, Wenting Yu, Peng Zhang, Zhi Huang, Anni Lin, Yan Xia

**Affiliations:** Academy of Electronic Engineering, Naval University of Engineering, Wuhan, China

## Abstract

In order to reach the intelligent recognition, the deep learning classifiers adopted by radar waveform are normally trained with transfer learning, where the pretrained convolutional neural network on an external large-scale classification dataset (e.g., ImageNet) is used as the backbone. Though transfer learning could effectively avoid overfitting, transferred models are usually redundant and might not generalize well. To eliminate the dependence on transfer learning and achieve high generalization ability, this paper introduced neural architecture search (NAS) to search the suitable classifier of radar waveforms for the first time. Firstly, one of the innovative technologies in NAS called differentiable architecture search (DARTS) was used to design the classifier for 15 kinds of low probability intercept radar waveforms automatically. Then, a method with an auxiliary classifier called flexible-DARTS was proposed. By adding an auxiliary classifier in the middle layer, the flexible-DARTS has a better performance in designing well-generalized classifiers than the standard DARTS. Finally, the performance of the classifier in practical application was compared with related work. Simulation proves that the model based on flexible-DARTS has a better performance, and the accuracy rate for 15 kinds of radar waveforms can reach 79.2% under the −9 dB SNR which proved the effectiveness of the method proposed in this paper for the recognition of radar waveforms.

## 1. Introduction

In modern electronic warfare, the classification of radar waveforms is one of the pivotal technologies in radar countermeasures and reconnaissance systems. It is also an important basis for judging the threat of enemy weapons [1, 2]. However, with the application of various new radar systems based on low probability of intercept (LPI) technology, traditional classification could not meet the needs of actual electronic warfare any more.

Researchers convert the waveform into two-dimensional time-frequency image by Choi–Williams distribution (CWD) time-frequency analysis [3] or other techniques and then send it to different models to achieve continuous upgrading of recognition capabilities. Due to the specific properties, different machine learning models can have different results even facing the same input [4]. Compared with other neural networks [5–7], the convolutional neural network (CNN) has a better performance in the processing of image, including radar and sonar images, facial images, and hand gesture images [8–10]. Therefore, it also has been widely used in the recognition of radar waveforms [7, 11–20].

There are two options for the CNN used in the research. First, according to different tasks, researchers design the CNN [14–17] independently. Kong et al. [14] take 12 kinds of radar waveforms as the target object and then debug the hyperparameters of the CNN repeatedly. After performing a lot of experiments, it achieved a better recognition accuracy than the same period model. However, designing a model from scratch requires researchers to try mistakes or set the parameters randomly. However, the performance may not be satisfactory. In order to avoid the tedious work manually, in recent years, people would like to choose the second option—transferring the CNN [21] that have been pretrained on external large-scale classification data (such as ImageNet [22]), LeNet [23], AlexNet [23–26], VGGNet [27], GoogLeNet [28–30], ResNet [28], DenseNet [31], and so on). In the latest study [31], researchers transferred the DenseNet as a classifier to reinforce the recognition accuracy in low signal-to-noise ratio (SNR). The accuracy of 8 kinds of waveforms can reach 93.4% at −8 dB SNR. However, Ghadimi et al. [30] pointed out that when they tried to transfer GoogLeNet which has been pretrained by almost 12 million images, evaluated by 50,000 images, and tested by 100,000 images, the differences between the pretrained datasets and the target datasets would increase the risk of overfitting too. The author faced the same tedious adjustment work when trying to transfer GoogLeNet for 9 kinds of radar waveforms. It can be seen from Table 1 that researchers have tried many ways to improve the accuracy rate of the radar waveforms. However, most of them only consider the accuracy of the classification algorithm and lack consideration of other performance indicators (such as model build time and misclassification rate) [35].

It can be seen that although transfer learning can solve the design problem of the model, there are two important issues that have been ignored. One is that the transferring model does not meet the requirements of transfer learning strictly. It is pretrained on an external large-scale optical image dataset which has a big gap to the radar waveform images obtained through time-frequency transformation; second, in order to have a better fitting ability to the huge dataset, the depth of the model is constantly deepening. However, it may be overfitting when faced to the smaller dataset such as radar waveform images. In general, it may not be the best choice.

To eliminate the dependence on transfer learning and achieve high generalization ability, we introduced the neural architecture search (NAS) [36] to the recognition of radar waveforms for the first time to design the classifier automatically. NAS is an algorithm that can automatically learn neural networks. It can design a network from the beginning which has the good performance so that it can be comparable to the expert level in some tasks [37]. By comparing the architecture search based on evolutionary algorithms [38] or reinforcement learning [39], we chose the differentiable neural network architecture search represented by differentiable architecture search (DARTS). DARTS turns the search space into a continuous space, has high search efficiency, and is the fastest search algorithm currently [40]. However, due to the approximate solution of the bilevel optimization problem, DARTS also faces difficulties such as unstable search results or performance degradation in the verification stage [41, 42]. In recent years, some improvement methods have also been explored [43, 44], but the methods were just suited for the specific tasks which could not be used generally. To solve the problem, we proposed a method with an auxiliary classifier (called flexible-DARTS) for architecture search which has the wide range for applications. By adding auxiliary classifiers in different output sizes of features, the improved method not only can reduce the structural difference between the search stage and the verification stage but also the optimization efficiency is higher as the propagation capability of loss value is stronger.

The main contributions of this paper can be summarized as follows:

(1). It is the first time to explore the method of improving radar signal waveform classification with the help of NAS
(2) To solve the problem of instability shown in DARTS, we propose a new method of architecture search with an auxiliary classifier called flexible-DARTS
(3) The two methods are verified on the experimental platform, and the data are compared with the previous research

The main structure of this article is as follows: Section 2 introduces DARTS and the flexible-DARTS proposed in this article and compares the performance of the two. Through experiments in Section 3, the excellent performance of the network architecture based on the flexible-DARTS is introduced, and its practicability to radar waveform recognition is also proved through the improvement of recognition accuracy rate. Finally, the conclusions of this paper are drawn in Section 4.

## 2. Methods

The concept that using DARTS to design the classifier for radar waveforms is firstly presented in part one. Besides, the inadequacy of DARTS is pointed out in part one too. Then, the flexible-DARTS with an auxiliary classifier proposed in this paper is offered in part two.

### 2.1. Standard DARTS

**Figure 1:**
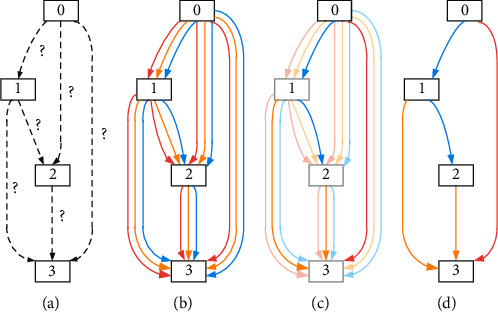
The process in DARTS search space.

DARTS obtains a cell through the training dataset, which is composed of input nodes, intermediate nodes, output nodes, and edges. Suppose each cell has two input nodes and one output node, for the convolutional network, the two input nodes are the output of the first two layers of cells. After multiple trainings, DARTS will form a large network. Hyperparameter can control the number of cells that connect to form the whole network. The whole process can be summarized: Figure 1(a) shows the initial form of the cell in the network, assuming that there are 4 nodes in a cell. In Figure 1(b), all the lines between nodes are connected. Between each two nodes is a mixed candidate operation, and each operation corresponds to a probability value. Figure 1(c) shows that, during the training process, the bilevel optimization problem is solved while optimizing the mixed probability and weight. Figure 1(d) shows that with the largest retention probability, the operation forms the final cell [40].

To make the search space continuous, we use softmax to relax the mixed weight of the operation. The specific scheme is detailed in [40]. The mixed operation between any set of nodes (*i*, *j*) is weighted by conditional probability as

(1) $$\begin{array}{c} {\bar{o}}^{\left(i,j\right)}\left(x\right)=\sum_{o\in O}\frac{\exp\left(\alpha_{o}^{\left(i,j\right)}\right)}{\sum_{o^{\prime }\in O}\exp\left(\alpha_{o^{\prime }}^{\left(i,j\right)}\right)}o\left(x\right). \end{array}$$

The conditional probability weight of the mixed operation is parameterized by the |*O*| dimensional vector *α*^(*i*, *j*)^. Through the model of formula (1), the problem of architecture search can be simplified to a learning problem of a set of continuous variables *α*={*α*^(*i*, *j*)^}. The process of solving the problem is shown in Figure 1. *L*_train_ represents the training set loss. *L*_val_ represents the validating set loss. After the operation is relaxed, the structural parameters *α* and weight *w* can be jointly learned. Similar to reinforcement learning or evolutionary algorithms, DARTS regards the performance of the verification set as the final reward or goodness of fit. The goal of DARTS is to minimize the loss of the verification set by using the gradient descent method to optimize.

**Figure 2:**
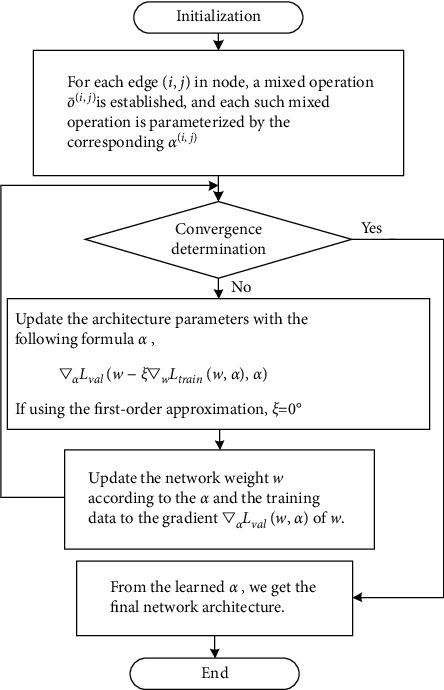
Workflow for optimizing *w* and *α*, using gradient descent.

A two-step method, adjusting *w* first and then adjusting *α*, and so on until convergence, is used in DARTS [40]. When the structural parameters of the outer layer change, the weight of the inner layer model must be recalculated. This process is very complex. Liu et al.[40] proposed an approximation scheme. The specific implementation algorithm for iterative optimization of *w* and *α* using gradient descent is shown in Figure 2.

According to the description, we can find that the updating process is the way to optimize *w* and *α* iteratively. The first-order approximation is a gradient descent optimization of the network weight *w*, whereas the second-order approximation means that when the gradient is updated on *α*, *w* is updated again, which makes *w∗*(*α*) definitely more accurate. In summary, the task of architecture search in DARTS can be summarized in two steps. The first step is to use DARTS for architecture search and to optimize the two types of computing units through the loss of the verification set; the second step is to build a network with optimized computing units, train on the training set from scratch, and validate its performance on the validating set. Although under the premise of gradient optimization, DARTS achieves excellent architecture search performance. However, there are still four problems when using DARTS for architecture search:

1. The search space of the differentiable architecture is insufficient, and the searchable architecture remains simple
2. Search results are unstable and easily affected by the initial values and the learning times
3. The consumption of hardware resources is still high
4. Performance may degrade when the architecture of the search is moved to validation sets

In order to reduce the adverse impact of the above contradictions shown in results, we proposed an improved search algorithm called flexible-DARTS. By adding an auxiliary classifier in the search stage, the flexible-DARTS has a better performance both in searching and validating.

### 2.2. The Proposal of Flexible DARTS

**Figure 3:**
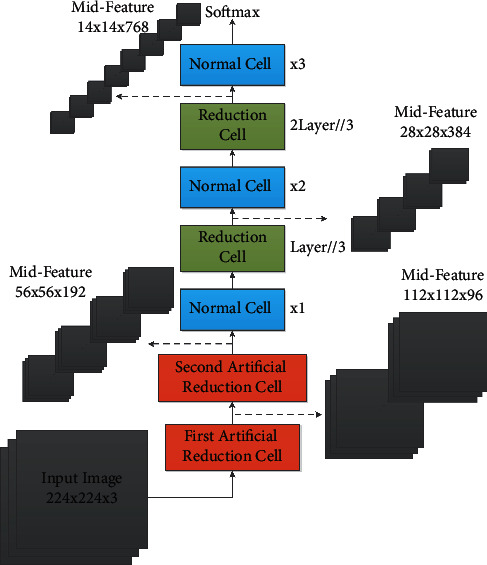
The workflow of standard DARTS during validating. The gray part represents the data or feature map, the green part represents the searched reduction unit, the blue part represents the searched standard unit, and the red part represents the hand-designed reduction unit.

As the fastest search algorithm up to now, DARTS always consumes a huge memory of the GPU during the search time. Sometimes, the ability of gradient backpropagation might be reduced. We referred to the NASNet experiment.  Figure 3 shows the application of the standard DARTS algorithm in the large-scale ImageNet. It could be seen that we needed to design two reduction levels manually to reduce the image size to 56 × 56 before we use the searched cells for classification tasks. When searching, DARTS has 8-level cells and does not set the auxiliary head. However, when it comes to verification, the number of cell levels might be increased to 20 (four intermediate nodes are set up for each level of cell). It is an obvious contradiction.

It can be seen that when the size of the data goes to be larger or the amount of training data becomes bigger, the performance of DARTS will face a big challenge. The network needs to be deeper to help extract better features, with the difficulty of searching becoming complex. Therefore, DARTS has chosen to adopt the plan which has been used in the GoogleNet called Inception. The problem of vanishing gradients can be solved by outputting additional features in the intermediate stage. It means that, in the architecture validation, the auxiliary classifier is introduced into the two-third level (when the feature map size is 8 × 8). However, in this case, DARTS uses auxiliary classifiers when validating but does not use auxiliary classifiers when searching, which might aggravate the structural difference between searching and validating (it also may reflect in the difference at the number of layers).

From this, we found that the standard DARTS has the following two directions to improve when searching, validating, and transferring to the target dataset. One is to shrink the structural difference during searching and validating, and the other is to reduce the manually designed network architecture. GoogleNet (also known as Inception V1) [45] research paper mentions: “On the classification task, the powerful performance of the shallower network shows that the features generated by the middle layer of the network are extremely discriminative.” By adding auxiliary classifiers to these middle layers, the discriminative power of the low-stage classifier can be improved, which not only overcomes the problem of gradient disappearance but also realizes the regularization. Therefore, GoogleNet uses a two-level auxiliary classification in the middle layer and adds two losses to overcome the disappearance of the gradient return. This can effectively reduce the disappearance of the gradient (the jump connection in ResNet is used to reduce the gradient explosion). However, experiments show that the influence of the auxiliary network is relatively small (about 0.5). It means that adding an auxiliary classifier during training can achieve the same effect.

**Figure 4:**
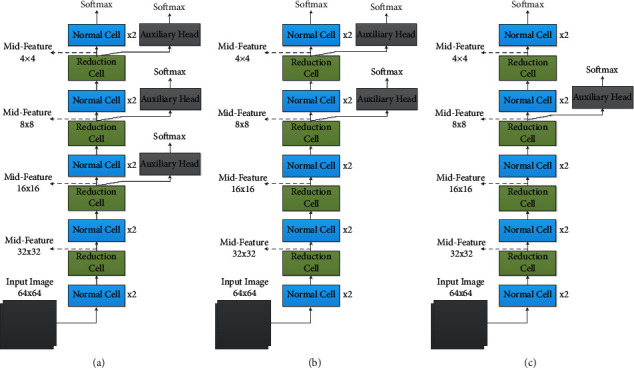
Three kinds of auxiliary classifier architecture. (a) Three auxiliary classifiers. (b) Two auxiliary classifiers. (c) One auxiliary classifier.

According to the abovementioned analysis, we proposed an algorithm, flexible-DARTS, which adopts an auxiliary classifier flexibly in searching time. Because of the manual part in the feature extraction of large-size images in DARTS, we discarded the manual part when facing large-size datasets. The cell architecture searched by flexible-DARTS was adopted in the whole process of feature extraction. In order to adapt to the requirements in architecture searching for the large-size image dataset, different search spaces have been used for the normal group and the reduction group. In addition, auxiliary classifiers have been added in the architecture to narrow the gap between the network architecture during searching and testing. In order to find the architecture with auxiliary classifiers which is the most suitable one for radar waveforms, we compared the performance in classification using different auxiliary classifiers. Three kinds of auxiliary classifier architecture are shown in Figure 4.

**Figure 5:**
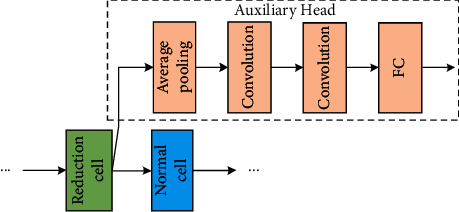
The architecture of the auxiliary classifier.

The architecture of the auxiliary classifier is described in Figure 5. It has four layers that contain one average pooling layer, two convolutional layers, and one fully connected layer.

## 3. Experiment

In this section, the experiment is divided into five parts. In part one, radar waveform datasets used in this study is introduced briefly. In part two, the model based on flexible-DARTS is compared with the model based on standard DARTS and the model based on 2CNN3 which is designed manually. In part three, the recognition capability of the model based on flexible-DARTS is discussed with related work. Besides, the confusion matrix is offered in part four to prove the experimental results are compared with Baidu EasyDL.

### 3.1. Dataset Representation

In the research, we have studied 15 kinds of waveforms, including LFM, NLFM, Costas, BPSK, five polyphase codes (including Frank, P1, P2, P3, and P4 codes), four multitime codes (including T1, T2, T3, and T4 codes), and two composite modulations (LFM/BPSK and 2FSK/BPSK), as shown in Table 2. On the assumption that the received signal would be interfered by the additive white Gaussian noise (AGWN), the carrier frequency has been regarded as the center frequency of the signal bandwidth in this paper. Therefore, the discrete-time sample model of the receiver output signal can be expressed as

(2) $$\begin{array}{c} y\left(k\right)=x\left(k\right)+w\left(k\right)=a\left(k\right)e^{j\theta \left(k\right)}+w\left(k\right), \end{array}$$

where *k* is the index value that sequentially increases with the sampling interval, *x*(*k*) is the ideal discrete signal after intermediate frequency sampling, *w*(*k*) is AGWN, and *a*(*k*) is the nonzero constant instantaneous signal envelope within the pulse interval. All the simulations in this article assigns *a*(*k*)=1. *θ*(*k*) is the instantaneous phase of the sampled signal, which can be expressed by instantaneous frequency *f*(*k*) and instantaneous phase offset *ϕ*(*k*):

(3) $$\begin{array}{c} \theta \left(k\right)=2\pi f\left(k\right)\left(kT_{s}\right)+\phi \left(k\right), \end{array}$$

where *T*_*s*_ is the sampling interval of the signal. In reality, we usually change the instantaneous frequency (frequency modulation) and instantaneous phase offset (phase modulation) of the signal to form different emission waveforms.

**Figure 6:**
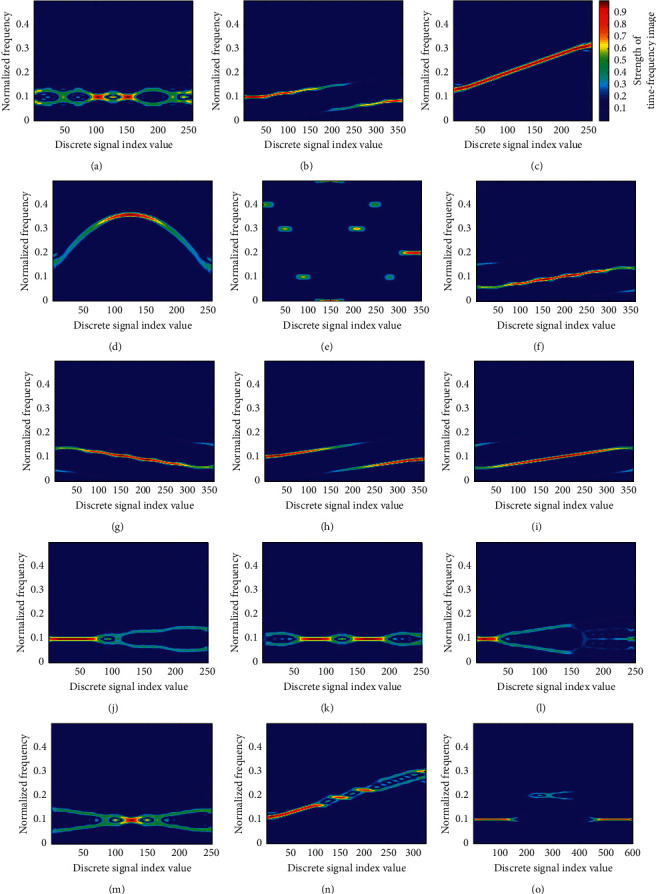
CWD time-frequency characteristic diagram of 15 typical radar signals given in Table 2 in a noise-free environment. (a) BPSK. (b) Frank. (c) LFM. (d) NLFM. (e) Costas. (f) P1. (g) P2. (h) P3. (i) P4. (j) T1. (k) T2. (l) T3. (m) T4. (n) LFM-BPSK. (o) 2FSK-BPSK.

In our research, the original image was converted into an image of size 64 × 64 by downsampling. On the premise of not losing too much information and meeting the needs of the classifier, we have reduced the consumption of the processor. The radar signals used in this paper were converted by CWD to obtain time-frequency images of fifteen types of signals in a noise-free environment, as shown in Figure 6.

### 3.2. Searching Results of the Two Algorithms in Methods

The dataset was generated in a simulation with 3 dB steps ranging from −9 dB to 9 dB. 15 kinds of signals had been generated to 800 samples, respectively, in different SNRs. Then, the samples generated above were allocated to the searching data and the validating data at a ratio of 3 : 1. Therefore, the searching data had 63,000 samples. The validating data had 21,000 samples. The model number of the CPU was Intel Xeon E5-2603. The model number of the GPU was Nvidia 1080Ti. The simulation framework was built by using Pytorch160.

Before the formal experiment, we used the three schemes in Figure 4 to search the architecture. After integrating the three indicators of video memory demand, search speed, and evaluation accuracy, the third improved architecture which was added an auxiliary branch architecture at the position of the 8 × 8 feature map shown in Figure 4(c) was finally decided to be used.

**Figure 7:**
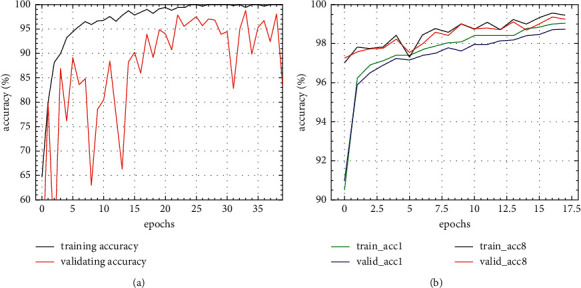
Performance curves during architecture searching. (a) Standard DARTS. (b) Flexible-DARTS.

The standard DARTS and flexible-DARTS were used to search the architecture for the searching data of the radar waveform. It would be stopped when the validating performance exceeds 99%. The search results on radar waveforms indicate that the FL-DARTS, where the hyperparameter is 1.2 MB, is powerful to design a better generalized classifier, of which the hyperparameter is about one-half of the DARTS-designed classifier where the hyperparameter is 2.3 MB. The performance curves during searching time are shown in Figure 7.

**Figure 8:**
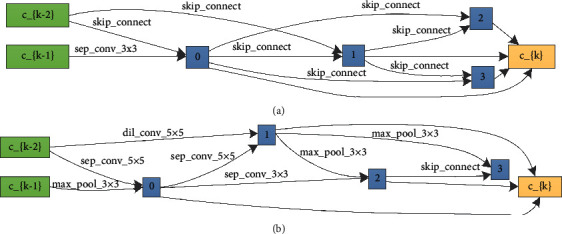
Cells searched on the radar signal dataset by the standard DARTS. (a) The searched normal cell. (b) The searched reduced cell.

**Figure 9:**
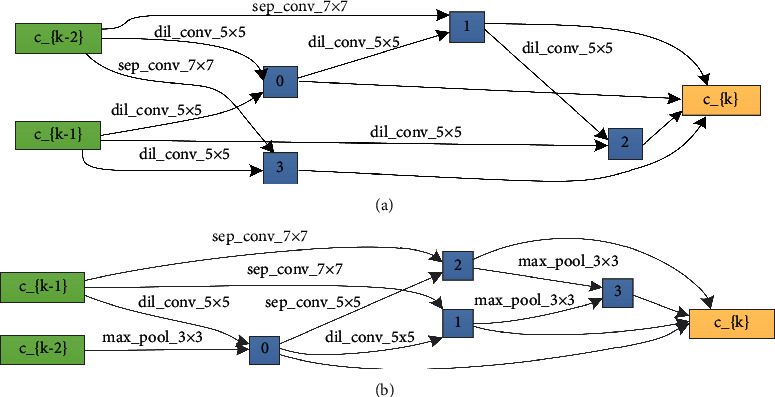
Cells searched on the radar signal dataset by the flexible-DARTS using the searched architecture shown in Figure 4(c). (a) The searched normal cell. (b) The searched reduced cell.

It can be seen from above figures that the flexible-DARTS is superior to the standard DARTS whether in searching speed or in training stability. The standard DARTS requires 38 epochs to complete the search even results might be very unstable. The flexible-DARTS only requires 17 epochs to complete the search, and the results are obviously stable. The searching results proves that the addition of the auxiliary classifier can enhance the stability of the search time. Besides, it can improve the searching efficiency and help to find the model with excellent performance. Figures 8 and 9 show the cells obtained by the standard DARTS and flexible-DARTS.

### 3.3. Comparison about the Classification Performance

**Figure 10:**
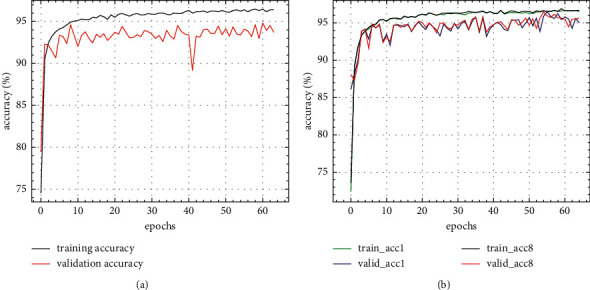
The performance during architecture training. (a) Standard DARTS. (b) Flexible-DARTS.

The cells shown in Figures 8 and 9 are the results of the searching part. After searching, it is time for them to be trained by the whole data. The performance during training is shown in Figure 10.

**Figure 11:**
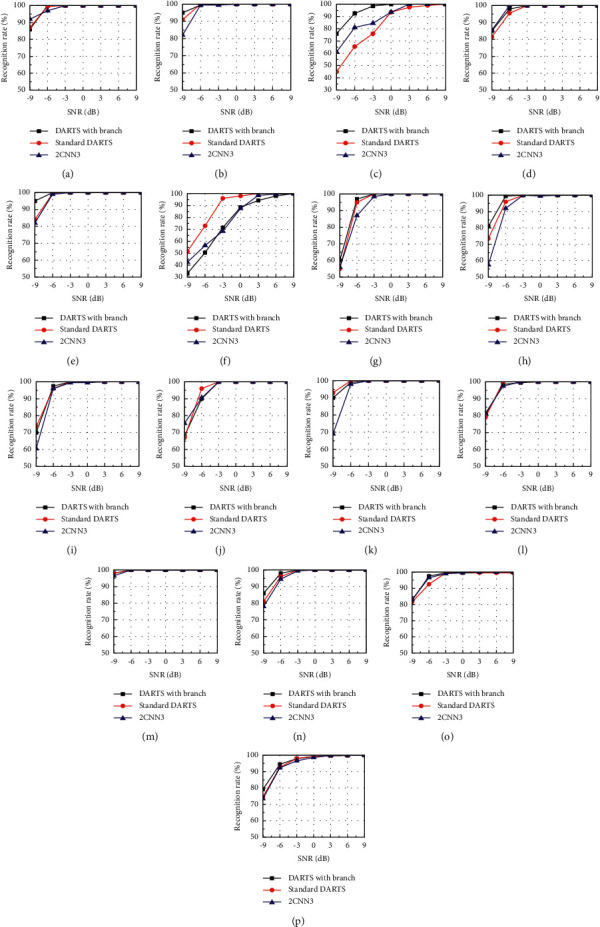
The comparison of recognition accuracy rates of the three methods. (a) BPSK. (b) Frank. (c) LFM. (d) NLFM. (e) Costas. (f) P1. (g) P2. (h) P3. (i) P4. (j) T1. (k) T2. (l) T3. (m) T4. (n) LFM-BPSK. (o) 2FSK-BPSK. (p) Overall.

We used standard DARTS, flexible-DARTS, and the previous research [46] (manually designed, represented by 2CNN3 which consists of four convolutional layers, four pooling layers, two fully connected layers, and one dropout layer, stride is 1) for validating. The results are shown in Figure 11.

It can be seen from Figure 11 that in terms of overall recognition accuracy rate, the flexible-DARTS is superior to the standard DARTS and 2CNN3. Under the −9 dB SNR, the DARTS with the auxiliary classifier proposed in this paper has a recognition accuracy rate of 79.2% for the 15 kinds of radar waveforms, which is about 5% higher than that of the standard DARTS 74.6% and 2CNN3 (73.5%). Compared with 2CNN3, the DARTS improves its recognition accuracy rate by 1% at −9 dB SNR. For Frank, P1, P3, T2, and LFM-BPSK signals, the recognition accuracy rate of the three shows the same trend as the overall recognition accuracy rate. The introduction of flexible-DARTS is higher than that of the standard DARTS and 2CNN3 which is the lowest. For P4 and T4, the standard DARTS has better performance under low SNR. For T1, T3, and LFM signals, the method of using the automatic search architecture is better than the manually designed network under low SNR. For BPSK, P2, T1, T4, Costas, and NLFM signals, the three methods have similar performance. In general, DARTS with the auxiliary classifier can achieve better recognition performance under low signal-to-noise ratio, which further proves the effectiveness of the method.

### 3.4. The Confusion Matrix about Radar Signal

**Figure 12:**
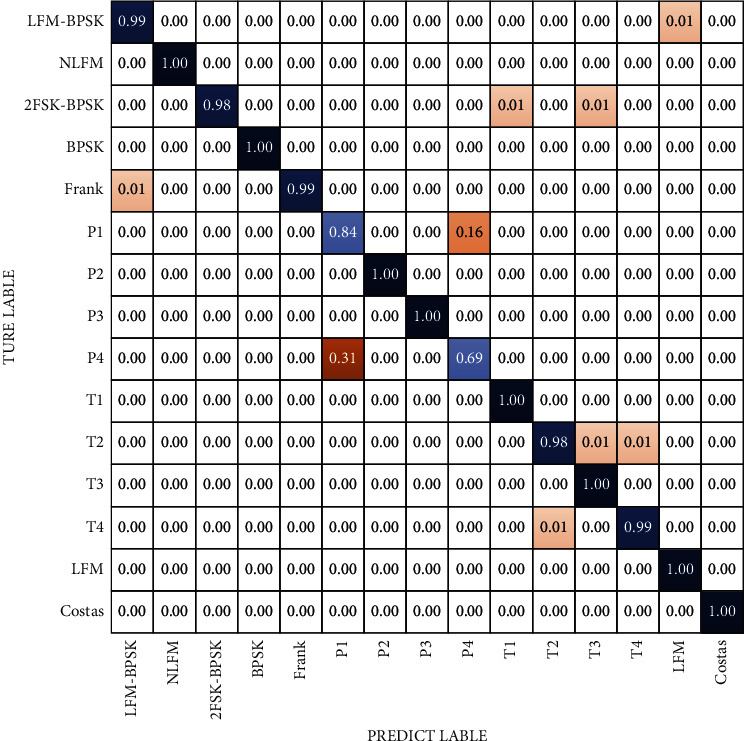
Confusion matrix of 15 typical radar signal waveforms in 2CNN3 at −3 dB.

**Figure 13:**
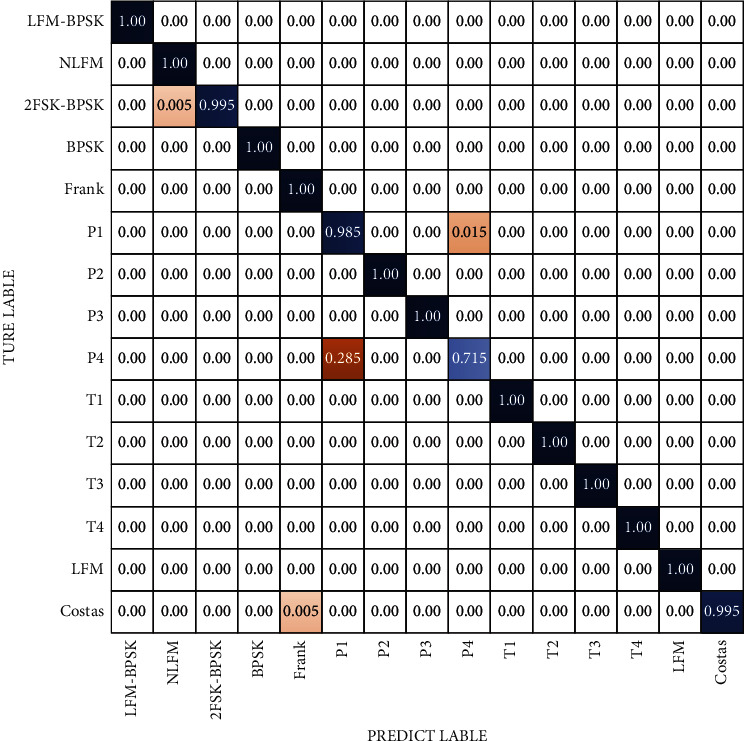
Confusion matrix of 15 typical radar signal waveforms in flexible-DARTS at −3 dB.

Previous research [18] found that, even if the network performance is good enough (it means that the network's recognition accuracy rate of the trained dataset had reached to a high level and the recognition accuracy rate to most of the waveforms can reach 99%), there are still some signals that are easily confused. The similarity between the waveforms is high (or the similarity between the converted time-frequency images is high) and the difference of the extracted features is not obvious. Confusion caused by the signal similarity is the main reason for classifier errors.  Figure 12 is the confusion matrix of 2CNN3. It can be found that under the training conditions of the dataset in this article, the characteristic images of the P1 signal and the P4 signal are very easily confused signals, and there is also a slight confusion between the T1 signal and the T3 signal.  Figure 13 is the confusion matrix of the recognition of each single signal. From the picture we can see that the anticonfused ability of the classifier based on the flexible-DARTS has been improved even in low SNR. The comparison shows that the flexible-DARTS has an excellent performance in improving the recognition of easily confused waveforms. For the easily confused P1 and P4, the recognition effect of P1 has been improved significantly, and the accuracy rate has increased from 84% to 98.5%, nearly 15%. Furthermore, there is no confusion between T1 and T3. However, the recognition accuracy rate for P4 which has only increased from 69% to 71.5% is still not ideal. Therefore, for radar waveform detection under low SNR, it is still necessary to adopt appropriate signal extraction methods to improve the recognition accuracy.

### 3.5. Comparison with Related Networks

**Figure 14:**
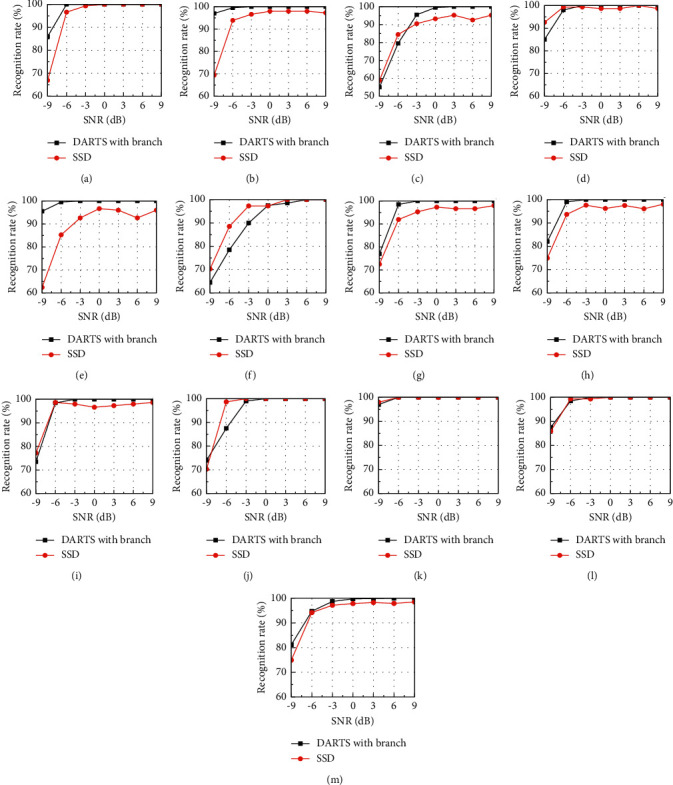
The comparison of recognition accuracy rates at three methods. (a) BPSK. (b) Frank. (c) P1. (d) P2. (e) P3. (f) P4. (g) T1. (h) T2. (i) T3. (j) T4. (k) LFM. (l) Costas. (m) Overall.

Linh et al. [47] used the single shot multibox detector (SSD) to generate multiple default candidate boxes to achieve a reasonable selection for the effective pixel area of the time-frequency image. When SSD retains the characteristics of the time-frequency image signal, the invalid pixels are eliminated, so that the results obtained are greatly improved when compared with the concurrent work. The datasets used in the literature [47] included 12 kinds of radar waveforms (BPSK, Frank, P1, P2, P3, P4, T1, T2, T3, T4, LFM, and Costas). The same dataset was produced through simulation from −9 dB to 9 dB with 3 dB steps. 12 kinds of signals had been generated to 800 samples, respectively, in different SNRs. The whole dataset has 67200 samples. Therefore, we compared the classification based on flexible-DARTS with the literature [47]. The simulation results of the recognition accuracy rate are shown in Figure 14.

It can be seen from Figure 14 that the classifier based on flexible-DARTS (referred to as flexible-DARTS) has a better performance than the SSD method (referred to as SSD) proposed in the literature [47]. The accuracy rate of the flexible-DARTS is higher than that of the SSD under each SNR especially under −9 dB SNR, where the overall accuracy rate of the flexible-DARTS which is higher than 80% is about 6% more than that of the SSD. Signal BPSK, Frank, P3, T1, and T2 have the same tendency with the overall accuracy rate. For P1, P2, and T3, although the accuracy rate of the flexible-DARTS is slightly lower at −9 dB SNR than that of the SSD, the performance would exceed significantly to SSD when the SNR is increasing. For P4 and T4, the performance of SSD is better. For LFM and Costas signals, the performance of the two classification networks is equivalent. It can be seen from Figure 14(m) that the overall recognition accuracy rate of the flexible-DARTS is better than that of the SSD.

**Figure 15:**
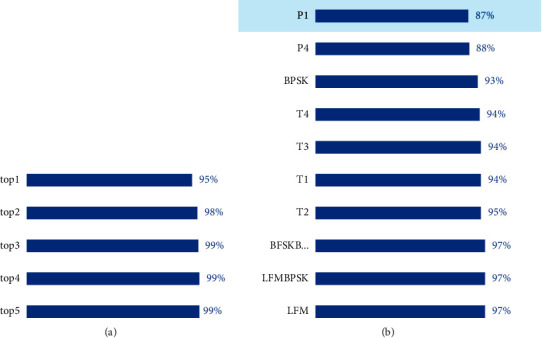
The EasyDL recognition results. (a) The comprehensive accuracy rate of EasyDL. (b) Main signal accuracy rate.

In addition to comparing with the abovementioned literature, our research was also compared with Baidu EasyDL. EasyDL is a customized AI training and service platform developed by Baidu Brain, which supports a one-stop AI development process from data management and data annotation, model training, and model deployment. Images, text, audio, video, and other data can be published to API, SDK, localized deployment, and software- and hardware-integrated products after EasyDL processing, learning, and deployment. The overall recognition result of EasyDL for the same dataset is shown in Figure 15. In the classification model evaluation report in Figure 15, the top 1–5 refers to the identification of data, and the model will give multiple results according to the level of confidence. Under normal circumstances, the recognition result with the highest confidence level is used, that is, the result of the top 1. As can be seen in the figure, the comprehensive accuracy rate of EasyDL classification results is 95%, which is lower than 95.89% of flexible-DARTS. Also, in the accuracy rate of a single signal, the flexible DARTS has a more excellent performance.

As a common platform, EasyDL can be transferred to solve most of the problems easily we met in our work. But from the results, it can be seen that transferring may not be the best choice when the requirement becomes more precise. As shown in Figure 15, it can be proved that the network obtained through automatic architecture search has more powerful feature extraction capabilities. The model designed for target datasets specifically shows an outstanding advantage even if the space becomes complicated.

## 4. Conclusion

In order to solve the dependence on transfer learning, this paper introduces neural architecture search into the recognition of radar waveforms, using differentiable architecture search (DARTS) to design the recognition model. Besides, in view of the unstable search results of DARTS and the performance degradation when validating, the difference of the model architecture between the search and validation has been studied. We proposed an optimized algorithm with the auxiliary classifier called flexible-DARTS. After comparing the performance of the multilevel auxiliary classifier by integrating the three indicators of model memory requirements, search speed, and evaluation accuracy, we decided to add an auxiliary classifier when the feature map is 8×8. Compared with the standard DARTS, the flexible-DARTS has an excellent stability when searching the model architecture. Besides, the search time of the flexible-DARTS is cut in half. Furthermore, the flexible-DARTS can help to find a model with powerful capabilities shown by the accuracy rate. The classifier of the 15 radar waveforms searched by the flexible-DARTS is about 5% higher than that of the standard DARTS at −9 dB SNR. In addition, we compared the network with other studies, including 2CNN3 [46] and classification based on SSD [47] and Baidu EasyDL. From the comprehensive recognition accuracy rate of all the results of 15 radar signals, the method in this paper is better than all of the three. The obvious increase in the resolution proves that the automatic architecture search can obtain a better-performing classifier. This shows that the transfer learning is not the best choice further, and the network matching the dataset obtained through the neural architecture search will have stronger practicality in the future. However, the improvement of the model performance based on the flexible-DARTS only depends on the improvement of the DARTS algorithm itself. It is due to the fact that it cannot find the exact location of the feature extraction, which makes it unable to integrate with other classification algorithms to improve its performance. It leads to a certain restriction on its future use.

## Figures and Tables

**Table 1:** The related works in classification of radar waveforms.

Type	Main idea
Improving in preprocess	Designing new features [19, 32]
	Improving the TFD algorithm [15, 33]
	Make the picture clearer [18, 29]

Improving the classifier	Structure expansion [6, 7, 16, 25]
	Designing the CNN manually [14–16]
	Replacing the fully connected layer (FC) with other structures [20, 24, 32]
	Transferring learning [23, 24, 26–31, 33, 34]

**Table 2:** 15 radar signal waveforms (mostly LPI).

Modulation type	*f*(*k*)	*ϕ*(*k*)
LFM	*f*_0_+*B*(*kT*_*s*_)/*τ*_pw_	Constant
NLFM	*f*_*c*_+*a*_1_(*kT*_*s*_)+*a*_2_(*kT*_*s*_)^2^	Constant
Costas	*f*_*j*_	Constant
BPSK	Constant	0, *π*
Frank	Constant	2*π*/*M*(*i* − 1)(*j* − 1)
P1	Constant	−*π*/*M*[(*M* − (2*j* − 1))][(*j* − 1)*M*+(*i* − 1)]
P2	Constant	−*π*/2*M*(2*i* − 1 − *M*)(2*j* − 1 − *M*)
P3	Constant	*π*/*ρ*(*i* − 1)^2^
P4	Constant	*π*/*ρ*(*i* − 1)^2^ − *π*(*i* − 1)
T1	Constant	2*π*/*N*_ps_⌊*N*_ps_/2*π*mod{2*π*/*N*_ps_⌊(*N*_si_(*kT*_*s*_) − *jτ*_pw_)*jN*_ps_/*τ*_pw_⌋, 2*π*}⌋
T2	Constant	2*π*/*N*_ps_⌊*N*_ps_/2*π*mod{2*π*/*N*_ps_⌊(*N*_si_(*kT*_*s*_) − *jτ*_pw_)(2*j* − *N*_si_+1/*τ*_pw_)*N*_ps_/2⌋, 2*π*}⌋
T3	Constant	2*π*/*N*_ps_⌊*N*_ps_/2*π*mod{2*π*/*N*_ps_⌊*N*_ps_*B*(*kT*_*s*_)^2^/2*τ*_pw_⌋, 2*π*}⌋
T4	Constant	2*π*/*N*_ps_⌊*N*_ps_/2*π*mod{2*π*/*N*_ps_⌊*N*_ps_*B*(*kT*_*s*_)^2^/2*τ*_pw_ − *N*_ps_*f*_*c*_(*kT*_*s*_)/2⌋, 2*π*}⌋
LFM-BPSK	*f*_*c*_+*B*/*τ*_pw_(*kT*_*s*_)	0, *π*
2FSK-BPSK	*f*_*ci*_	0, *π*

*Note*. mod{*a*, *b*} is the remainder between *a* and *b*. ⌊*α*⌋ is the largest integer less than or equal to *α*. *M* and *ρ* are the number of encoding phase, but the difference is that *ρ* has to take the ability that can open square values. *i* and *j* are the iterative integer values from 1 to *M*. *N*_ps_ is the number of phase state. *N*_si_ is the number of step frequencies. *f*_*c*_ is the fixed carrier frequency value. *f*_*n*_, *f*_*m*_, and *f*_*ci*_, respectively, represent different frequency jump sequences of corresponding signals, where *n*=1,2,…,5, *m*=1,2,…, 6, and *i*=1,2. *τ*_pw_ is the pulse width. *a*_1_ and *a*_2_ are constants.

## Data Availability

Previously reported (python program) data were used to support this study and are available at arXiv:1806.09055.
